# Splenic irradiation before allogeneic stem cell transplantation for myelofibrosis

**DOI:** 10.1007/s12032-019-1245-5

**Published:** 2019-01-08

**Authors:** Grzegorz Helbig, Agata Wieczorkiewicz-Kabut, Mirosław Markiewicz, Helena Krzemień, Michał Wójciak, Krzysztof Białas, Małgorzata Kopera, Ewa Rzenno, Krzysztof Woźniczka, Anna Kopińska, Iwona Grygoruk-Wiśniowska, Anna Koclęga

**Affiliations:** 1 Department of Hematology and Bone Marrow Transplantation, School of Medicine in Katowice, Medical University of Silesia, Dąbrowski Street 25, 40-032 Katowice, Poland; 2 Students’ Research Group, Medical University of Warsaw, Warsaw, Poland

**Keywords:** Myelofibrosis, Splenomegaly, Splenectomy, Splenic irradiation, Allogeneic stem cell transplantation, Outcome

## Abstract

Splenectomy before allogeneic stem cell transplantation (ASCT) for patients with myelofibrosis (MF) remains a matter of debate, and conflicting results have been reported to date. The procedure seems to fasten post-transplant hematological recovery, but it does not have an impact on survival. The role of pre-transplant splenic irradiation (SI) is much more difficult to evaluate. Forty-four patients (25 males and 19 females) with MF at median age of 49 years at diagnosis (range 14–67) underwent ASCT. The post-transplant outcome was compared between irradiated and non-irradiated patients. Eleven patients received irradiation before transplantation. Median dose of radiation was 1000 cGy (range 600–2400). There was no difference in median time to engraftment between patients with and without previous radiotherapy. Acute and chronic graft versus host disease (GVHD) occurred in 47% and 36% of patients, respectively. There was no difference in GVHD incidence between groups. Eight patients relapsed/progressed in irradiated group versus 17 in non-irradiated (70% vs. 51%; *p* = 0.3). Transformation to acute myeloid leukemia was observed in 3 patients: 2 in irradiated and 1 in non-irradiated group. In total, 22 patients died with no statistical difference in death rate between irradiated and non-irradiated subjects. The probability of overall survival after transplant for the entire cohort at 2 years was 54% (72% for irradiated and 48% for non-irradiated patients; *p* = 0.25). Splenic irradiation prior to ASCT for myelofibrosis has not beneficial effect on post-transplant outcome.

## Introduction

Myelofibrosis (MF) is a BCR-ABL-negative myeloproliferative neoplasm associated with bone marrow fibrosis and extramedullary hematopoiesis. Most patients carry JAK2, CALR or MPL mutation; however, none of these abnormalities are specific and essential for diagnosis of MF. Clinical manifestations of MF are variable; one-third of patents are asymptomatic at presentation, the remaining have symptoms resulting mainly from anemia and splenomegaly (SM) [1]. The latter finding is a consequence of extramedullary hematopoiesis. SM is found in 90% of MF patients at initial presentation, and its size increases with disease duration producing pain in left upper quadrant and early satiety [2].

To date, only allogeneic stem cell transplantation (ASCT) remains the only curative option for patients with MF. However, due to high transplant-related mortality, ASCT is limited to small proportion of MF patients, usually for those at younger age at transplant, without significant co-morbidities and at higher prognostic categories [3, 4].

A pre-transplant splenectomy remains a matter of debate, and conflicting results have been reported to date. The procedure seems to fasten post-transplant hematological recovery, but its impact on relapse rate and survival is unclear. A trend for faster leukocyte engraftment but a significant higher incidence of relapse at 3 years was demonstrated for splenectomized MF patients in a large analysis of EBMT group. Of note is, that splenectomy had no impact on overall survival [3]. A French Study has confirmed the beneficial effect of splenectomy on post-transplant engraftment. Moreover, splenectomy in men had favorable impact on overall survival [5]. Pre-transplant splenectomy in MF patients was not associated with post-transplant relapse but with a prolonged overall and event-free survival in a recent published study [6].

The role of pre-transplant splenic irradiation (SI) in MF patients is much more difficult to evaluate. It was demonstrated in patients transplanted for chronic myeloid leukemia that SI before ASCT did not increase relapse incidence and transplant-related mortality [7]. There are only few small studies on SI prior to ASCT in MF patients [8, 9].

In this paper, we report on the effects of pre-transplant SI on outcome of MF patients undergoing ASCT.

## Materials and methods

The study patients were identified through the use of our institutional database of medical records. The diagnosis of MF was established according to WHO criteria [10]. Data on cytogenetic results were not reported as dry aspirates were present in a majority of patients at MF diagnosis, and thus, metaphases were not obtained. Risk stratification was performed according to the Dynamic International Prognostic Scoring System (DIPSS) [11]. Spleen size before and after ASCT was assessed using palpation and abdominal ultrasound. SI was indicated for patients with markedly enlarged splenomegaly at the discretion of treating physician. Doses and fractionation of splenic irradiation was individually based. All patients provided an informed consent in accordance with the Declaration of Helsinki.

Nonparametric comparisons of group means were performed by using the Mann–Whitney *U* test. Proportions were compared by Fisher exact test. The distribution for overall survival (OS) was estimated using the method of Kaplan and Meier and compared using the log-rank test. A *p* value less than 0.05 was considered significant. All computations were performed with StatSoft Poland analysis software (version 10.0).

## Results

### Patient characteristics

Forty-four patients (25 males and 19 females) with MF at median age of 49 years at diagnosis (range 14–67) underwent ASCT between 2004 and 2017. The diagnosis was as follows: primary myelofibrosis (PMF; *n* = 30), post-polycythemia vera myelofibrosis (post-PV MF; *n* = 10) and post-essential thrombocythemia myelofibrosis (post-ET MF; *n* = 4). According to the DIPSS, 3 patients were stratified to low risk group, 3 to intermediate-1, 8 to intermediate-2 and 29 to high. The *JAK2* V617F point mutation was detected in 29 patients (66%), 12 patients were negative, and data were missing for one. 50% of studied patients were red blood cells (RBCs) transfusion-dependent at the time of diagnosis. Median number of treatment lines was 1 (range 0–5). Most frequent pre-transplant treatments included hydroxyurea (*n* = 23), corticosteroids (*n* = 15), thalidomide (*n* = 7), interferon α (*n* = 5), androgens (*n* = 5). The other therapeutic options included anagrelide (*n* = 4), ruxolitinib (*n* = 2) and intensive chemotherapy (*n* = 2).

Eleven patients received splenic radiotherapy before transplant. Median time between SI and ASCT was 1.5 months (range 0.5–12). Median dose of radiation was 1000 cGy (range 600–2400) administered in a median of 8 fractions (range 6–15). There was tendency to greater spleen size in irradiated patients (*p* = 0.06). Decrease in spleen size was demonstrated in 4 out of 11 (36%) patients after SI. No severe complications of SI were observed. Patients’ characteristics at diagnosis according to previous radiotherapy are shown in Table 1.

**Table 1:** Comparison of patient characteristics at diagnosis according to previous radiotherapy

Parameter	With radiotherapy	Without radiotherapy	*p* value
Number of patients	11	33	
Gender (female/male)	5/6	14/19	0.5
Age (median, range); years	49 (33–67)	49 (14–67)	0.8
Primary disease (*n*; %)			0.39
Primary MF	6 (55)	24 (73)	
Post-PV MF	3 (27)	7 (21)	
Post-ET MF	2 (18)	2 (6)	
IPSS (*n*; %)			0.14
Low	0	3 (9)	
Intermediate-1	0	3 (9)	
Intermediate-2	2 (18)	6 (18)	
High	9 (82)	20 (60)	
*JAK2* V617F mutation (*n*; %)			0.59
Positive	9 (82)	22 (67)	
Negative	2 (18)	10 (30)	
Missing	0	1 (3)	
Spleen size below costal margin (cm)	10 (5–16)	9 (0–20)	0.38
RBCs dependence (*n*; %)			0.46
Yes	5 (45)	17 (52)	
No	6 (55)	15 (45)	
Missing	0	1 (3)	
Number of treatment lines before transplant	1 (0–5)	1 (0–3)	0.5

Median time from diagnosis to transplant was significantly shorter for patients without pre-transplant splenic irradiation when compared with those who underwent radiotherapy: 41 months (range 2–643) versus 122 months (18–210); *p* = 0.02.

### Transplant data

Median age at transplant was comparable between two studied groups (51 vs. 52 years; *p* = 0.7). Blood parameters did not differ except hemoglobin level which was statistically higher for patients undergoing radiotherapy. Dynamic IPSS was intermediate-2 or high in 84% of transplanted patients. Approximately 50% of patients remained RBCs and platelets transfusion-dependent at the time of transplantation. Constitutional symptoms were present in 20 out of 44 included subjects. Despite previous splenic radiotherapy, the pre-transplant spleen size measured on ultrasound was comparable between two groups of analyzed patients (*p* = 0.9). HLA identical sibling was available for 19 patients, the remaining received transplant from matched unrelated donors. Peripheral blood was a source of stem cells for all but one patient who received bone marrow. Reduced intensity conditioning consisted of busulphan and fludarabine was used in all but one patient who was treated with myeloablative regimen (busulphan and cyclophosphamide). Anti-thymocyte globulin (ATG) was administered in patients transplanted from unrelated donors. Graft versus host disease (GHVD) prophylaxis was uniform and included cyclosporine and methotrexate. One exception was a patient with mild renal failure at transplant who received cyclosporine with mycophenolate. Median number of transplanted CD34-positive cells were comparable between irradiated and non-irradiated subjects.

All patients but one with early post-transplant death, engrafted and there was no difference in median time to engraftment between patients with and without previous radiotherapy. Only one death within the first 30 days after transplant was noted and it occurred in non-irradiated patient. In total, eight patients did not achieve platelet count > 20 × 10^9^/L at the day of discharge.

More than 50% of all patients demonstrated infectious complications after transplant; however, there was no difference between irradiated and non-irradiated subjects. The most frequent infectious manifestations included pneumonia (*n* = 9), urinary tract infection (*n* = 7), bacteremia (*n* = 5), pneumocystis pneumonia (*n* = 4), pharyngitis (*n* = 3), diarrhea (C. difficile; *n* = 2). CMV reactivation was observed in three patients. Three patients developed septic shock successfully treated with antibiotics. Fever of unknown origin was seen in four patients.

Acute and chronic GVHD occurred in 47% and 36% of patients, respectively. Acute GVHD grade III/IV was present in 2 irradiated and 1 non-irradiated patients. There was no difference in GVHD occurrence between both groups. Twenty-five patients developed disease relapse or progression and three of them received second or subsequent transplant. Eight patients relapsed/progressed in irradiated group versus 17 in non-irradiated (70% vs. 51%; *p* = 0.3).

Transformation to acute myeloid leukemia was observed in three patients: two in irradiated and one in non-irradiated group.

In total, twenty-two patients died (4 in irradiated and 18 in non-irradiated). There was no statistical difference in death rate between irradiated and non-irradiated subjects (63% vs. 45%; *p* = 0.48). One patient died within the first 30 days after transplant due to cerebral hemorrhage, six patients expired between + 30 and + 100 days after transplant and the remaining died at later stage.

Among four irradiated patients who expired, two died of severe GVHD with subsequent infectious complications (bacterial and viral). Chemotherapy-resistant acute myeloid leukemia developed in the remaining two subjects. In non-irradiated subgroup, all but one patient died of bilateral pneumonia (bacterial or fungal) associated with immunosuppressant-resistant GVHD and CMV reactivation.

Twenty-two patients (50%) are alive at the last contact. Twenty subjects were found to have full donor chimerism, and results were not available for additional two. Median time from diagnosis to last contact reached 4.5 years (range 0.6–22.3), and median time from transplant to last contact was 9.9 months (range 0.7–113). The results were comparable between analyzed groups. Transplant data are summarized in Table 2.

**Table 2:** Transplant data in irradiated and non-irradiated patients with myelofibrosis

Parameter	With radiotherapy	Without radiotherapy	*p* value
Age (median, range); years	51 (38–68)	52 (22–69)	0.7
WBC count (× 10^9^/L); median, range	9.63 (1.28–198.9)	7.1 (0.8–222.5)	0.47
Hemoglobin (g/dL); median, range	10.4 (8.3–15.6)	8.9 (5.9–16.5)	0.05
Platelet count (10^9^/L); median, range	130 (18–690)	97 (16–674)	0.1
DIPSS (*n*; %)			0.18
Low	0	3 (9)	
Intermediate-1	0	3 (9)	
Intermediate-2	1 (9)	5 (15)	
High	10 (91)	21 (67)	
PLT transfusion-dependence (*n*; %)			0.38
Yes	3 (27)	12 (36)	
No	8 (63)	19 (54)	
RBCs transfusion-dependence (*n*)			0.36
Yes	5 (45)	19 (63)	
No	6 (55)	14 (37)	
Constitutional symptoms (*n*; %)			0.59
Yes	5 (45)	15 (45)	
No	6 (55)	18 (55)	
Spleen size below costal margin (cm) before transplant	16 (12–24)	8 (0–18)	0.06
Spleen size below costal margin (cm) on day + 30 after transplant	5 (0–14)	4 (0–20)	0.7
Type of donor (*n*; %)			0.05
HLA identical sibling	2 (18)	17 (52)	
HLA matched unrelated	9 (82)	16 (48)	
Number of transplanted CD34-positive cells; median, range	5.9 (1.25–8.32)	6.5 (1.08–13.01)	0.32
ANC > 0.5 (× 10^9^/L); median, range	15 (13–18)	15 (11–29)	0.7
PLT > 20 (× 10^9^/L); median, range	26 (10–47)	18 (10–67)	0.14
Acute GVHD (*n*; %)	5/11 (45)	16/33 (48)	0.5
Chronic GVHD (*n*; %)	3/8 (37)	5/9 (45)	0.64
Infectious complications (*n*; %)	4 (36)	21 (63)	0.1
Relapse/progression (*n*; %)	8 (70)	17 (51)	0.3
Alive (*n*; %)	7 (63)	15 (45)	0.48
Median time from transplant to last contact (months); median, range	11.5 (5.9–86.9)	10 (0.7–113)	0.6
Median time from diagnosis to last contact (years); median, range	5.4 (0.9–12.5)	3.9 (0.6–22.3)	0.42

**Fig. 1:**
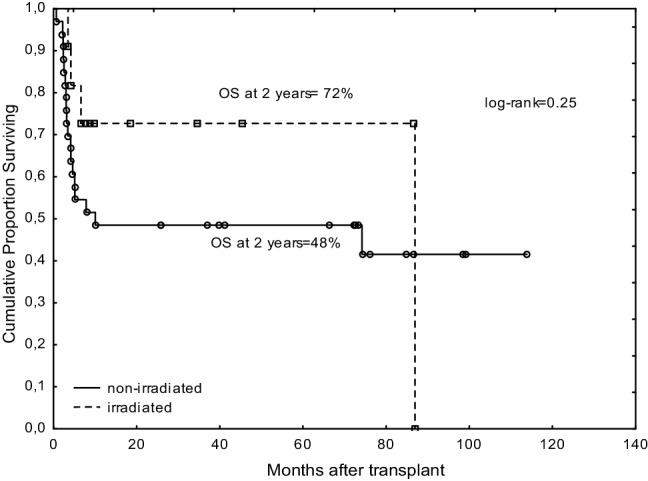
Comparison of overall survivals between irradiated and non-irradiated patients with myelofibrosis

The probability of overall survival after transplant for the entire cohort at 2 years was 54% (72% for irradiated and 48% for non-irradiated patients; *p* = 0.25) (Fig. 1).

## Discussion

The presence of extensive splenomegaly in MF patients undergoing ASCT delays post-transplant granulocyte recovery when compared with those who had splenectomy [12]. The question on the role of splenectomy before ASCT remains open. One should bear in mind an increased mortality and morbidity of splenectomy versus faster post-transplant engraftment. However, in the era of JAK2 inhibitors, the question should soon be unfounded.

The role of splenic irradiation in patients with MF is not well established. It was demonstrated that SI alleviated splenic discomfort and reduced spleen size in a majority of MF patients. Moreover, SI was associated with stabilization or increase of body weight. The median duration of response was 6 months, with maximum duration of response exceeded 41 months. The life-threatening myelosuppression was present in 26% of the patients and half of them expired [13]. The risk of severe and durable complications of SI remains the main limitation of this procedure, and nowadays it is not routinely advised in daily clinical practice. Total doses of SI are variable and range from 150 to 6500 cGy per course administered in a fractionated manner [2]. Interestingly, lower radiation doses (100 cGy in 4 daily doses) administered as an induction-maintenance treatment resulted not only in splenomegaly reduction but also in circulating blast clearance in two patients [14].

Data on the effect of spleen status on outcome after ASCT for myeloproliferative and myelodysplastic disorders have been collected by the Center for International Blood and Marrow Transplant Research (CIBMTR) group. The authors analyzed allografts from a large number of transplanted patients including those with prior splenectomy and splenic irradiation. It was demonstrated that splenectomized patients had faster neutrophil and platelet engraftments when compared with non-splenectomized patients without having an impact on survival. The incidence of acute and chronic GVHD did not differ between irradiated, splenectomized and non-splenectomized patients. Overall mortality rates were also comparable across the groups. However, one should keep in mind that study population was heterogeneous and dose and timing of SI differ between patients. All this may cause difficulties in data interpretation [15].

Taking into account the rarity of data on SI prior to ASCT in MF, we compared the post-transplant outcome between irradiated and non-irradiated patients. We did not observe any difference in engraftment rates, acute and chronic GVHD incidences and survivals between those two analyzed groups. Our study population was homogeneous in terms of diagnosis, conditioning and GVHD prophylaxis. However, there were limitations associated with variations in radiation doses and timing before ASCT. Splenic irradiation is known to result in prolonged pancytopenia with infectious complications, however, that was not a case in our group. A proportion of patients has already been pancytopenic at the time of SI and the median time between SI and ASCT was relatively short (1.5 month). Nevertheless, the incidence of post-transplant infectious complications was comparable between analyzed groups.

The role of SI in leukemic transformation (LT) remains unclear and speculative. It is difficult to conclude whether LT results from prior radiation or represents natural disease course. No difference in LT was present between irradiated and non-irradiated patients in our study.

Reports on the role of SI prior to ASCT for myelofibrosis are sparse and included small groups of patients or case studies [8, 9, 16, 17]. Eight patients with MF received median splenic radiation dose of 450 cGy within median of 2 weeks prior to ASCT in Kalman study [8]. All patients had massive pre-transplant splenomegaly (> 20 cm) and achieved a significant decrease in spleen size during repeated assessments. The toxicity of SI was mild. Post-transplant recovery, disease-free and overall survivals were comparable with those who were not irradiated. These results were then confirmed by others. Administration of SI as a part of reduced-intensity conditioning at doses ranging from 300 to 500 cGy in three patients was found to be feasible with acceptable toxicity [9].

## Conclusions

Despite favorable safety profile, splenic irradiation prior to ASCT for myelofibrosis has not beneficial effect on post-transplant outcome and should not be routinely recommended.
